# HIV-1 envelope replication and α4β7 utilization among newly infected subjects and their corresponding heterosexual partners

**DOI:** 10.1186/1742-4690-10-162

**Published:** 2013-12-26

**Authors:** Victor Pena-Cruz, Behzad Etemad, Nikolaos Chatziandreou, Phyu Hninn Nyein, Shannon Stock, Steven J Reynolds, Oliver Laeyendecker, Ronald H Gray, David Serwadda, Sandra J Lee, Thomas C Quinn, Manish Sagar

**Affiliations:** 1 Department of Medicine, Division of Infectious Diseases, Boston University, Boston, MA, USA; 2 Department of Mathematics and Computer Science, College of the Holy Cross, Worcester, MA, USA; 3 Department of Medicine, Johns Hopkins University School of Medicine, Baltimore, MD, USA; 4 Division of Intramural Research, National Institute of Allergy and Infectious Diseases, National Institutes of Health, Bethesda, MD, USA; 5 Department of Epidemiology, Bloomberg School of Public Health, Johns Hopkins University, Baltimore, MD, USA; 6 Makerere University School of Public Health, Kampala, Uganda; 7 Departments of Biostatistics and Computational Biology, Dana Farber Cancer Center, Boston, MA, USA; 8 Boston University, Evans Biomedical Research Building, 650 Albany Street, Room 647, Boston, MA 02118-2518, USA

**Keywords:** HIV-1, Envelope, Transmission, Receptor, Replication, Alpha4 beta7, Dendritic cells, Langerhans cells, Selection

## Abstract

**Background:**

Previous studies suggest that active selection limits the number of HIV-1 variants acquired by a newly infected individual from the diverse variants circulating in the transmitting partner. We compared HIV-1 envelopes from 9 newly infected subjects and their linked transmitting partner to explore potential mechanisms for selection.

**Results:**

Recipient virus envelopes had significant genotypic differences compared to those present in the transmitting partner. Recombinant viruses incorporating pools of recipient and transmitter envelopes showed no significant difference in their sensitivity to receptor and fusion inhibitors, suggesting they had relatively similar entry capacity in the presence of low CD4 and CCR5 levels. Aggregate results in primary cells from up to 4 different blood or skin donors showed that viruses with envelopes from the transmitting partner as compared to recipient envelopes replicated more efficiently in CD4+ T cells, monocyte derived dendritic cell (MDDC) – CD4+ T cell co-cultures, Langerhans cells (LCs) – CD4+ T cell co-cultures and CD4+ T cells expressing high levels of the gut homing receptor, α4β7, and demonstrated greater binding to α4β7 high / CD8+ T cells. These transmitter versus recipient envelope virus phenotypic differences, however, were not always consistent among the primary cells from all the different blood or skin donation volunteers.

**Conclusion:**

Although genotypically unique variants are present in newly infected individuals compared to the diverse swarm circulating in the chronically infected transmitting partner, replication in potential early target cells and receptor utilization either do not completely dictate this genetic selection, or these potential transmission phenotypes are lost very soon after HIV-1 acquisition.

## Background

Landmark studies more than 20 years ago demonstrated that newly infected subjects often harbor a limited number of HIV-1 variants early after virus acquisition [1-3]. Subsequent studies further showed that naïve individuals are often infected with a single or multiple variants, and the complexity of the early virus population is influenced by factors present at the time of acquisition, such as genital tract inflammation [4-7]. More recent studies have robustly estimated both the number and the characteristics of the strains present early after infection [8-14]. In aggregate, these diverse studies demonstrate that even though chronically infected individuals harbor a large array of variants, only a small number of viruses with specific characteristics are able to successfully establish a persistent systemic infection in a naïve host.

The biological mechanisms underlying this observed bottleneck during transmission remain undefined. Because the acquired viruses in the newly infected subject often do not cluster among the major variants present in the transmitting partner, stochastic mechanisms likely do not account for the genetic restriction observed during transmission [15]. Genotypic and phenotypic studies show that the acquired variants predominantly utilize the CCR5 coreceptor and contain signature envelope genotypes, such as shorter and less glycosylated envelope variable loops that are more closely related to ancestral strains [11-13,16-20]. In aggregate, these studies suggest that specific viruses with unique characteristics are favored for transmission from the quasispecies present in the transmitting partner.

Identifying the virus property that confers fitness during transmission has been a high priority within the field because this understanding may foster the development of targeted interventions to prevent acquisition. Because variants with shorter and less glycosylated envelopes were enriched among the virus populations sampled early after infection, it was hypothesized that transmitted viruses may have more exposed receptor binding sites leading to enhanced receptor utilization and higher replication capacity. Envelopes from viruses found early after infection or the inferred transmitted/founder (T/F) viruses, however, have not demonstrated an enhanced ability to utilize low CD4 or CCR5 levels or a higher capacity to enter cells compared to the envelopes from the variants present in the corresponding transmitting partner or those present during the chronic stage of disease [21-26]. These previous studies have often used virus pseudotypes to investigate potential transmission phenotypes. Pseudoviruses cannot be used to probe virus replication in the target cells present at the site of invasion as a potential phenotype that confers fitness during transmission. One recent study showed that full-length T/F strains replicated significantly more efficiently compared to unrelated chronic stage variants [27]. Selection of a relatively small number of unrelated chronic stage variants, however, may have biased this comparison. Thus, the previous studies have not adequately examined replication capacity differences in potential early target cells among envelopes isolated from transmission linked partners.

Besides infection capacity in early target cells, a variants’ ability to disseminate from the initial site of invasion could also potentially influence the observed genetic restriction during HIV-1 acquisition. After HIV-1 establishes a beachhead in a new host, the virus cannot be detected in the systemic circulation for a number of days [28-30]. During this silent phase, the virus presumably replicates at the local site of invasion and then migrates to gut associated lymphoid tissue (GALT). Systemic dissemination early after acquisition is associated with high level replication within GALT [31]. Newer studies speculate that binding to the α4β7 integrin may play a crucial role in the migration of the virus from the exposure site to GALT [32,33]. Interestingly, HIV-1 envelope glycoprotein subunits with characteristics associated with newly acquired viruses demonstrate high binding to α4β7+ cells, and this attachment decreased with envelope modifications observed over the course of infection suggesting that this may be a highly transient transmission phenotype [34].

In this study, we generated replication competent recombinant viruses incorporating pooled HIV-1 envelopes isolated from 9 recently infected individuals and their corresponding heterosexual partner in Rakai, Uganda. We compared replication in potential early target cells, coreceptor tropism, receptor utilization efficiency, and fusion capacity among viruses with HIV-1 envelope glycoproteins isolated from these transmission pairs. By comparing genotypic and phenotypic features among viruses found in newly infected subjects compared to those present in the transmitting partner, our studies provide new insights for the biological mechanisms for the genetic selection during transmission.

## Results

### Couples and envelope sequences

We retrospectively identified 8 couples from the Rakai Couple Cohort Study (RCCS) in which the newly infected subject was sampled prior to HIV-1 seroconversion. We were successfully able to amplify full-length envelopes from 9 of the 16 individuals in these partnerships. In 2 couples envelopes were amplified from both the newly infected recipient and the transmitting partner, and in 5 couples envelopes were successfully generated from only 1 of the 2 partners. Surprisingly, we were unable to amplify full-length envelopes in five seronegative subjects even though they had HIV-1 RNA levels greater than 100,000 copies/ml. To increase the number of couples, we also retrospectively identified 12 other couples in whom the newly infected subject was sampled within a year after estimated infection. Transmitter and recipient envelopes were successfully amplified from both partners in 8 couples. In the remaining partnerships, envelopes were either amplified from 1 of the 2 partners (n = 3) or in none of the individuals (n = 1). Various different primer combinations failed to yield full-length envelope PCR product in the unsuccessful cases.

**Figure 1:**
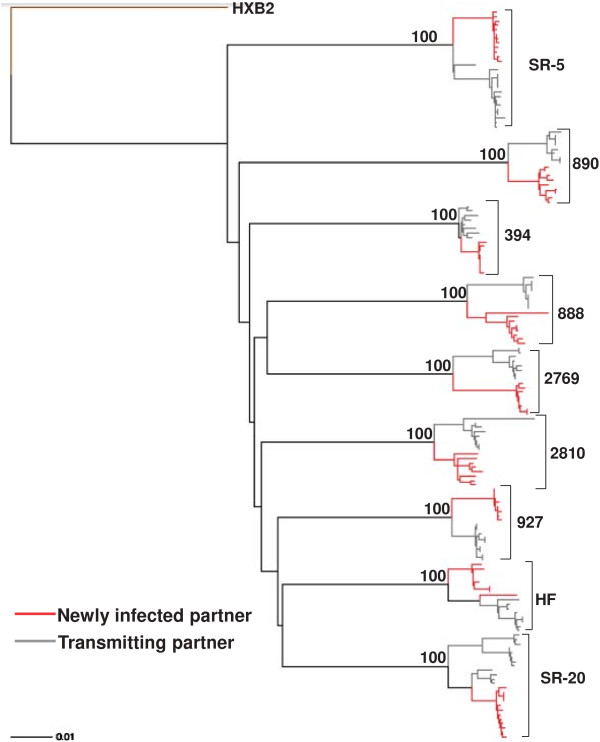
**Epidemiologically linked partner’s HIV-1 envelope sequences are phylogenetically linked.** Full-length HIV-1 envelope transmitter (gray) and recipient (red) sequences were aligned with subtype reference sequences from the Los Alamos database using Clustal X. Paup was used to generate the maximum likelihood tree using parameters from FindModel best fit evolutionary model. Bootstrap values from 100 replicates were generated from a neighbor joining tree and are noted on each node of interest. Couple IDs and HXB2 reference outgroup node are noted.

Amplified product from a minimum of 4 independent bulk PCRs were pooled to minimize resampling bias [35]. Pooled envelope products were cloned into a HIV-1 NL4-3 backbone using yeast gap-repair homologous recombination [36]. From each subject, full-length envelope sequences were examined from 8 to 12 different clones. Phylogenetic analysis incorporating reference sequences and other previously isolated full-length envelope sequences from the RCCS confirmed the epidemiological partnership in 9 of the 10 couples (Figure 1). Recipient and transmitter sequences failed to cluster in one of the epidemiologically linked couples suggesting that the newly infected partner acquired HIV-1 from outside the partnership (data not shown). Further genotypic and phenotypic analysis was continued in 9 couples with the confirmed sequence linkage (Table 1) (Figure 1). The 9 couples examined in this study were all infected with subtype D HIV-1. The newly infected partner in these nine couples was sampled a median of 70 days (range 17 – 324 days) after estimated infection. Longitudinal follow up in the Rakai cohort suggested that the transmitting partner had been infected for a minimum of 2 years prior to transmission to the newly infected recipient. Concurrent samples were obtained from each partner a median of 19 days (range 0 – 46 days) apart. If early host pressure selects against viruses harboring a property that confers fitness for transmission, we hypothesized that variants isolated within 3 months to 1 year after estimated acquisition should be relatively similar to the viruses circulating in the chronically infected transmitter. Thus, observing differences among recipient transmitter envelope properties could still yield important information even though the majority of the recently infected individuals were not sampled relatively soon after estimated acquisition.

**Table 1:** Demographics, viral and coreceptor characteristics

**Couple**	**Type**^**1**^	**Int. days**^**2**^	**Partner interval**^**3**^	**Recipient CCR5**^**4**^	**Recipient CXCR4**^**5**^	**Recipient tropism**^**6**^	**Transmitter CCR5**	**Transmitter CXCR4**	**Transmitter tropism**
HF	FTM	17	3	7.24	<0.1	R5	8.27	<0.1	R5
888	MTF	74	19	10.39	<0.1	R5	11.91	<0.1	R5
890	MTF	138	12	3.79	<0.1	R5	2.27	<0.1	R5
394	MTF	93	2	7.79	<0.1	R5	10.85	<0.1	R5
927	MTF	324	46	12.55	<0.1	R5	13.37	<0.1	R5
2769	MTF	149	46	5.69	<0.1	R5	5.34	0.65	R5/X4
2810	MTF	161	23	5.49	<0.1	R5	6.12	<0.1	R5
SR-5	MTF	17	0	12.62	<0.1	R5	9.72	<0.1	R5
SR-20	MTF	91	34	6.70	<0.1	R5	7.24	<0.1	R5

^1^FTM: female to male; MTF: male to female.

^2^Interval in days from the estimated date of acquisition to the day of sample collection for the newly infected partner. For seronegative individuals, interval was estimated as a maximum of 17 days.

^3^Interval in days between sampling of the two partners within a couple.

^4^P24 (ug/ml) from U87/CD4/CCR5 cells at day 4 post-infection.

^5^ P24 (ug/ml) from U87/CD4/CCR5 cells at day 4 post-infection.

^6^Tropism as determined on U87/CD4+/CCR5 and U87/CD4+/CXCR4 cells.

In thirteen couples from the RCCS, we had previously shown that a limited number of minority variants closely related to the ancestral sequences were preferentially acquired by a naïve subject from the variants present in the transmitter [12]. Adding the nine couples to the previous thirteen reported partnerships, we confirmed that sequences present in the newly infected subject compared to those in the transmitter had a significantly shorter distance to the estimated most recent common ancestor (MRCA) (median ratio of recipient versus transmitter sequences’ distance to MRCA 0.82, range 0.32 – 1.42, p < 0.001). Recipient sequences as compared to transmitter envelopes were also less genetically diverse (median ratio of recipient versus transmitter genetic diversity range 0.57, range 0.11 – 5.69, p < 0.001) and were significantly less divergent (median ratio of recipient versus transmitter genetic divergence 0.39, range 0.06 – 9.89, p < 0.001). Recipient compared to transmitter envelopes also had significantly lower number of amino acids in the V1-V2 ((median 66 range (57 – 78) versus 68 (59 – 77) p = 0.014)), V1-V4 ((median 282 range (268 – 297) versus 284 (266 – 298) p = 0.001)) and V1-V5 ((median 364 range (322 – 392) versus 368 (323 – 393) p < 0.001)). Similar to the previous results from thirteen couples, there was no significant difference in predicted Asparagine (N)-linked glycosylation sites (PNGS) among the recipient and transmitter sequences in the more comprehensive analysis of the 22 couples [12]. In addition, envelopes from the newly infected individual had significantly lower V3 loop charge (median 4, range 2–7) compared to the transmitting partners sequences (median 4, range 2–9, p < 0.001). Within each couple, there were multiple amino acids that were present at different frequencies among the recipient compared to the transmitter envelopes, but the previously identified signature pattern at HBX2 position 12 of the signal peptide was not consistently different among the partners [10]. In addition, a PNGS at HXB2 position 413–415 was also not overrepresented in the transmitting as compared to recipient partners’ envelopes as described in the analysis of subtype B HIV-1 early and chronic infection sequences [10]. In aggregate, the analysis with the larger number of couples confirmed our previous finding that shorter less charged envelopes more closely related to estimated ancestral sequences were enriched during the early period after HIV-1 acquisition [12,19].

### Replication competent recombinant viruses and coreceptor tropism

Previous studies have primarily examined HIV-1 envelope glycoprotein properties using 293T derived virus pseudotypes capable of a single infection cycle. We produced peripheral blood mononuclear cell (PBMC) derived virus stocks to generate replication competent viruses. Each virus stock contained pooled envelopes from a minimum of three independent cloning attempts and was generated from passage on PBMCs from 5 different donors. We confirmed that the short passage PBMC virus stocks contained similar level of envelope genotypic diversity as evident in the original clones and did not demonstrate selection sweep (Additional file 1: Figure S1). Virus titers were not significantly different among recipient (median 930 infectious particle (IP)/ul, range 15 – 18,933 IP/ul) compared to transmitter enveloped viruses (median 1233 IP/ul, range 16 – 13,200 IP/ul, p = 0.8). Increased V3 loop charge as observed in the transmitter in comparison to the recipient envelopes has been associated with CXCR4 usage [37,38]. All recipient and the majority of transmitter viruses utilized the CCR5 and not the CXCR4 receptor (Table 1).

### Sensitivity to CD4 and CCR5 receptor and fusion blockers

**Figure 2:**
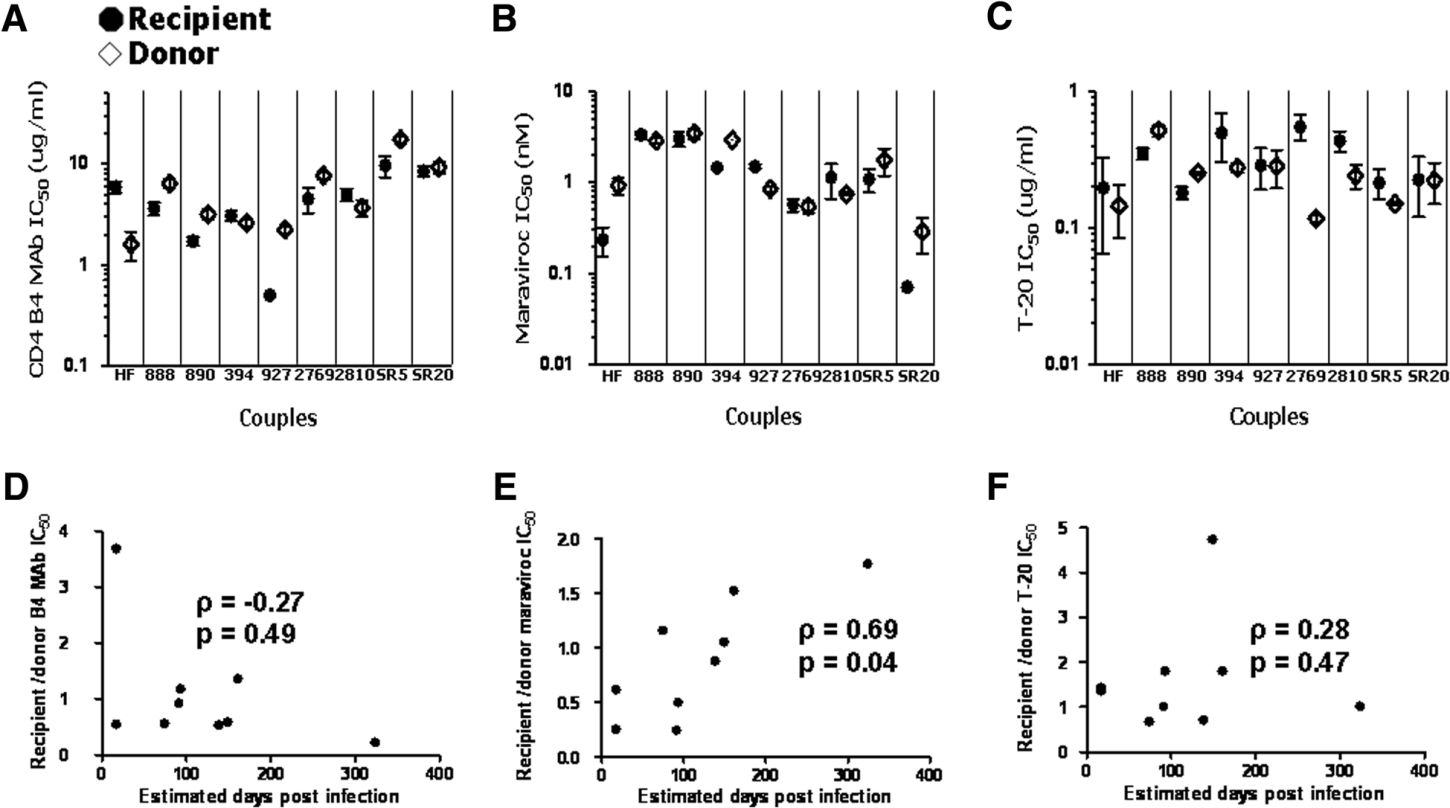
**Recipient and transmitter envelope viruses have no significant differences in sensitivity to receptor and fusion blockers.** IC_50_s to CD4 antibody **(A)**, Maraviroc **(B)**, and T-20 **(C)** among recipient (hollow circle) and transmitter (filled rectangle) envelope viruses. The x-axis shows the couple ID. Values represent means from a minimum of 3 independent experiments with error bars showing the standard deviation. Correlation between interval from estimated infection to sampling and recipient to transmitter IC_50_ ratios to CD4 antibody **(D)**, Maraviroc **(E)**, and T-20 **(F)**. Each graph shows a correlation coefficient with a Spearman rank correlation p - value.

Target cells at the site of invasion potentially have low cell surface CD4 and CCR5 concentrations, and viruses with an enhanced ability to infect these cells may have an advantage during transmission [39]. We and others have shown that sensitivity to receptor blockers correlates with a virus’ ability to replicate in cells with limiting receptor levels [24,40]. Viruses with a capacity to infect cells that have low receptor levels demonstrate high inhibitor IC_50_s, while variants that require high CD4 or CCR5 show low IC_50_s against the receptor blocker. We measured sensitivity to CD4 monoclonal antibody (MAb), B4, as a surrogate for CD4 utilization [41]. All viruses were inhibited by more than 50% at the highest B4 concentration, 50 ug/ml. Recipient IC_50_s ranged from 0.5 – 9.6 ug/ml while transmitter IC_50_s varied from 1.6 – 17.5 ug/ml (Figure 2A). In 6 of the 9 couples, transmitter as compared to the corresponding recipient envelope viruses displayed higher CD4 B4 MAb IC_50_ suggesting that transmitter viruses had a greater ability to utilize low CD4 receptor levels. Aggregate pair-wise comparison, however, showed no significant differences among recipient versus transmitter envelope virus sensitivity to CD4 B4 MAb (p = 0.2).

Because newly infected subjects were sampled at various times after estimated acquisition, the isolated envelopes potentially had genetic changes that modified their phenotypic properties compared to those present in the infecting strains. To assess this possibility, we examined the correlation between the recipient versus transmitter ratio for a phenotype of interest and the duration between estimated acquisition and sampling of the newly infected subject (referred to as time post infection). A transient transmission associated phenotype would potentially display a negative linear, exponential, or polynomial relationship with time post infection. In these cases, the recipient to transmitter ratio is higher in couples where the newly infected subject was sampled relatively early after acquisition, and the ratio decreases as time post infection increases. There was a negative correlation between the ratio of recipient to transmitter B4 MAb IC_50_ and estimated days post infection (ρ = -0.27, p = 0.49), but it was not statistically significant (Figure 2D). Furthermore, the goodness of the fit was not significantly higher assuming either a polynomial or exponential decay (data not shown).

We examined sensitivity to CCR5 antagonist, Maraviroc (MVC), as a surrogate measure for the ability to enter cells expressing low CCR5 receptor concentrations. All viruses were inhibited by more than 90% at the highest MVC concentration of 25 nM. Recipient IC_50_s ranged from 0.1 – 3.3 nM while transmitter IC_50_s varied form 0.2 – 3.5 nM (Figure 2B). In aggregate, there was no significant difference in MVC sensitivity among two groups of viruses (p = 0.4). There was, however, a significant positive correlation between the recipient to transmitter ratio of Maraviroc IC_50_ and estimated days post infection (ρ = 0.69, p = 0.04) (Figure 2E). The significant positive correlation suggests that compared to the corresponding transmitting partner’s envelopes the MVC IC_50_ is lower in newly infected subjects sampled relatively early after acquisition compared to those isolated later in infection. This observation supports ours and others previous findings that viruses found early in infection require higher amounts of the CCR5 receptor for cell entry compared to those present during the chronic phase of disease [24,36,42-46].

After receptor engagement, virus entry depends on fusion kinetics. Previous studies have shown that sensitivity to fusion blocker, T-20, directly correlates with fusion kinetics [47,48]. Highest T-20 concentration (10 ug/ml) produced around 100% cell entry block among all viruses. T-20 IC_50_s ranges were similar among recipient (range 0.2 – 0.6 ug/ml) and transmitter (range 0.1 – 0.5 ug/ml) envelope viruses. In 7 of the 9 couples, recipient as compared to the corresponding transmitter envelope viruses displayed higher T-20 IC_50_ suggesting that viruses found in newly infected subjects had enhanced fusion, but these differences were not statistically significant (p = 0.2) (Figure 2C). In addition, recipient to transmitter T-20 IC_50_ ratio did not demonstrate a significant negative correlation with days from acquisition (ρ = 0.28, p = 0.47) (Figure 2F).

### Replication in primary peripheral blood mononuclear cells

**Figure 3:**
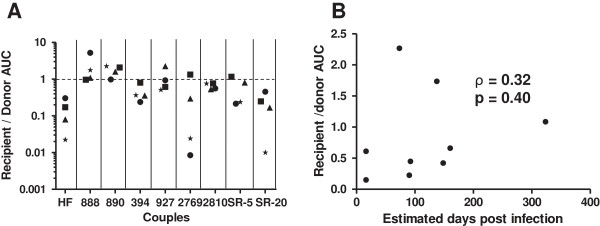
**Transmitter envelope viruses replicate significantly better than recipient envelope viruses in CD4+ T cells. (A)** Recipient relative to transmitter envelope virus replication area under the curve (AUC) among CD4+ T cells from blood donation volunteer 1 (circle), 2 (square), 3 (triangle), and 4 (star). X-axis shows the couple’s ID. The dotted line at a ratio of 1 separates instances when the recipient envelope viruses replicated to a greater extent than the transmitter envelope viruses (> 1) or vice-versa (< 1). **(B)** Correlation between interval from estimated infection to sampling and recipient to transmitter CD4+ T cell replication AUC ratio. Graph also shows the correlation coefficient with a Spearman rank correlation p - value.

Replication differences in early target cells potentially influences which virus establishes a new infection within a naïve host. We compared replication among viruses with recipient versus transmitter envelopes in activated CD4+ T cells from 4 different blood donation volunteers. There was large variation in AUC between the different blood donor’s cells suggesting that different blood donation volunteers CD4+ T cells supported replication to varying levels (Additional file 1: Figure S2). First, results from each blood donation volunteer’s cells were analyzed independently. Recipient and transmitter envelope viruses displayed no statistically significant replication differences (p > 0.05) in any of the 4 blood donation volunteer’s CD4+ T cells. Next, we conducted an aggregate examination of newly infected subject’s virus AUC relative to the corresponding transmitting partner’s virus AUC (Figure 3A). The median recipient to transmitter envelope virus AUC ratio in the CD4+ T cells from 4 different blood donation volunteers was 0.55 (range 0.01 – 5.21). A value below 1 indicates that the transmitter envelope virus replicated to higher level compared to the corresponding recipient envelope variants. Although, in 5 of the 9 pairs, the recipient as compared to the transmitter virus replicated better in at least one blood donation volunteer’s CD4+ T cells, in aggregate, transmitter envelope viruses were significantly better at replicating in activated CD4+ T cells compared to recipient envelope viruses (p = 0.03). The interval between sampling and estimated acquisition did not significantly correlate with recipient to transmitter AUC ratio (ρ = 0.32, p = 0.40), suggesting that this difference was relatively stable (Figure 3B).

### Replication in dendritic cells with and without autologous T cells

Intact mucosa prevents direct access to CD4+ T cells because these cells are mostly present in deeper sub-mucosal locations [49-52]. Cells of the monocyte lineage, such as DCs, are thought to provide a conduit for the virus to sub-epithelial CD4+ T cells [53]. We compared replication among recipient and transmitter envelope viruses in MDDCs in the absence or presence of autologous activated CD4+ T cells. First, we independently examined replication in immature and mature MDDC exposed to infectious virus. None of the 18 different virus stocks replicated in immature and mature MDDCs from 2 of the 3 blood donation volunteers. In one blood donation volunteer’s immature and mature MDDCs, low level replication was observed among a small number of transmitter and recipient envelope viruses (Additional file 1: Figure S3). These results confirmed that both immature and mature DCs are rarely productively infected when exposed to low levels of infectious virus potentially because of low CD4 and CCR5 surface receptor levels and potent anti-viral responses [39,54].

**Figure 4:**
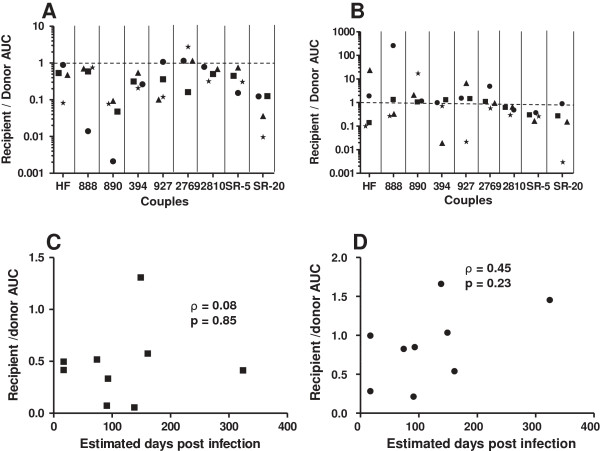
**Transmitter envelope viruses have higher replication compared to recipient envelope viruses in monocyte derived dendritic cells (MDDC) – autologous CD4+ T cell cultures.** Recipient relative to transmitter envelope virus replication AUC among mature MDDCs – autologous CD4+ T cells **(A)** and immature MDDCs – autologous CD4+ T cells **(B)** from blood donation volunteer 1 (circle), 2 (square), 3 (triangle), and 4 (star) respectively. The x-axis shows the couple’s ID. The dotted line at a ratio of 1 separates instances when the recipient envelope viruses replicated to a greater extent than the transmitter envelope viruses (> 1) or vice-versa (< 1). The bottom graphs show correlation between interval from estimated infection to sampling and recipient to transmitter replication AUC ratio in mature MDDC **(C)** and immature MDDC **(D)**. Graphs also shows the correlation coefficient with the Spearman rank correlation p - values.

Next, we examined replication in MDDC – autologous CD4+ T cell co-cultures. Replication levels varied between the different blood donation volunteer’s immature and mature MDDCs – CD4+ T cell co-cultures (Additional file 1: Figure S4). Transmitter as compared to recipient envelope viruses replicated significantly more (p < 0.05) in each of the 4 different blood donation volunteer’s mature DC - autologous CD4+ T cell co-cultures. In aggregate, the transmitter envelope viruses replicated to higher level compared to the corresponding recipient envelope variants (median 0.32, range 0.009 – 2.77, p < 0.001) (Figure 4A). On the other hand, recipient and transmitter envelope viruses displayed no statistically significant replication differences (p > 0.05) in any of the 4 blood donor’s immature DC - CD4+ T cells co-cultures. In aggregate, transmitter envelope viruses were better at replicating in immature MDDC – CD4+ T cell cultures than recipient envelope variants (median recipient to transmitter AUC 0.71, range 0.003 – 253.9), although this was not statistically significant (p = 0.30) (Figure 4B). The interval between sampling and estimated acquisition did not significantly correlate with recipient to transmitter AUC ratio for either mature (ρ = 0.08, p = 0.85) (Figure 4C) or immature MDDC (ρ = 0.45, p = 0.23) (Figure 4D).

### Replication in skin derived Langerhans cells heterologous T cell co-cultures

**Figure 5:**
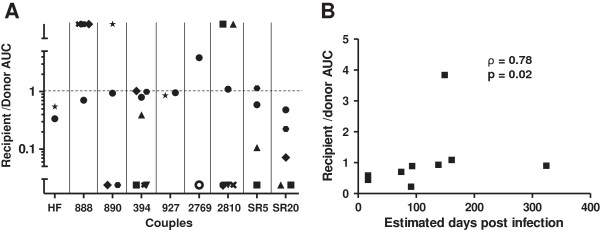
**Transmitter as compared to recipient envelope viruses replicate to higher levels in Langerhans cells (LCs) – heterologous activated CD4+ T cell cultures. (A)** Recipient relative to transmitter envelope virus replication AUC among the 10 different skin donation volunteers LCs. Each symbol represents LCs from a different subject. The x-axis shows the couple’s ID. The dotted line at a ratio of 1 separates instances when the recipient envelope viruses replicated to a greater extent than the transmitter envelope viruses (> 1) or vice-versa (< 1). Symbols in the bottom and top third of the y-axis depict instances where replication was observed either only in the recipient or transmitter envelope virus within a partnership respectively. **(B)** Correlation between interval from estimated infection to sampling and recipient to transmitter replication AUC ratio. Graphs also shows the correlation coefficient with the Spearman rank correlation p - values.

Genital mucosa contain specific tissue resident DCs, termed LCs [55,56]. Because LCs project dendrites over the lumen, they are likely the first DC subset that encounters incoming HIV-1. We compared replication differences among recipient versus transmitter envelope viruses in these cells [49,50]. LCs were isolated from anonymous discarded skin obtained from reduction mammoplasties using previously described methods [57,58]. More than 90% of the isolated cells expressed langerin and CD1a, a hallmark of LCs (Additional file 1: Figure S5). Similar to the MDDCs, virus failed to replicate in skin LC cultures alone but did propagate in LC - activated CD4+ T cell co-cultures. All viruses replicated in 1 donor’s LCs – CD4+ T cell co-cultures, but replication was observed among some of the recipient and transmitter envelope viruses in the remaining co-cultures with LCs from 9 other skin tissue donors. In the 1 donor’s LCs that supported replication of all variants, higher amounts of infectious virus was observed in transmitter as compared to recipient envelope viruses in 7 of the 9 couples, and this difference was marginally significant (p = 0.05). In aggregate, the transmitter envelope viruses replicated to higher level compared to the corresponding recipient envelope variants (median recipient to transmitter AUC 0.75, range 0.07 – 3.83, p = 0.02) among the cases where viruses from both partners in a relationship demonstrated replication (Figure 5A). In 19 instances virus from only 1 of the partners in a relationship (13 transmitter and 6 recipient) replicated in the LC – CD4+ T cell co-cultures. The replication AUC ratio between recipient and transmitter envelope viruses increased with the interval of time between estimated infection and sampling (ρ = 0.78, p = 0.02) (Figure 5B). This suggests that the recipient envelope viruses most closely related to the potential infecting strains (i.e. those sampled earliest after estimated HIV-1 acquisition) demonstrated the least efficient replication in LC – heterologous CD4+ T cells cultures compared to the corresponding transmitting partner’s envelope variants.

### Alpha4beta7 integrin usage

**Figure 6:**
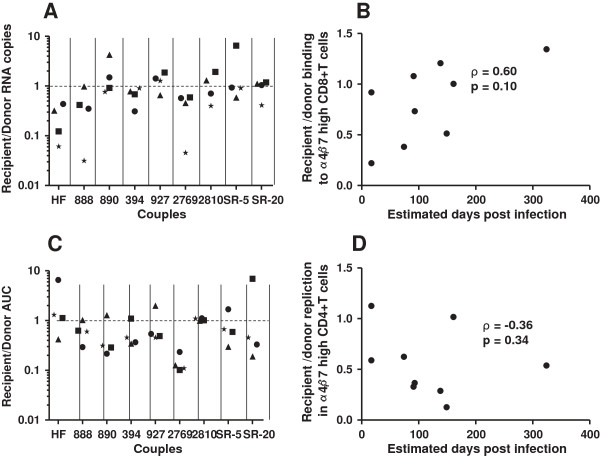
**Transmitter envelope viruses demonstrate higher α4β7 usage.** Graph A and C show recipient versus transmitter HIV-1 RNA copies recovered from α4β7 high / CD8+ T cells **(A)** and ratio of recipient to transmitter infectious virus concentration in the culture supernatant 3 days post-infection of α4β7 high / CD4+ T cells **(C)**. The x-axis shows the couple’s ID. The dotted line at a ratio of 1 separates instances when the recipient envelope viruses demonstrated greater binding or replication relative to the transmitter envelope viruses (> 1) or vice-versa (< 1). Graph B and D show correlation between interval from estimated infection to sampling and recipient to transmitter α4β7 high CD8+ T cell binding ratio **(B)** and α4β7 high CD4+ T cell replication ratio **(D)**. Graphs also shows the correlation coefficient with the Spearman rank correlation p - value.

It has been suggested that viruses with enhanced binding to the gut homing integrin, α4β7, are more likely to disseminate from the initial site of invasion to GALT, which is potentially important for establishing a systemic infection [32-34]. Previous studies from our group and others have shown that α4β7 inhibitors often fail to prevent virus binding to and replication in cells expressing high levels of α4β7 [26,59]. Thus, retinoic acid (RA) stimulated CD8+ and CD4+ T cells with flow cytometry confirmed up-regulated α4β7 expression were used to compare integrin binding and replication among recipient and transmitter envelopes in the absence of inhibitors (Additional file 1: Figure S6) [32,33]. Amount of HIV-1 RNA bound to α4β7 high CD8+ T cells was used to estimate binding to the gut homing integrin. One of the 4 blood donor’s cells demonstrated significantly greater transmitter as compared to recipient envelope virus binding to α4β7 CD8+ T cells (p = 0.03). In aggregate, transmitter relative to recipient envelope viruses demonstrated significantly higher binding to α4β7 high CD8+ T cells (median recipient to transmitter ratio 0.74, range 0.03 – 6.49, p = 0.04) (Figure 6A). In addition, there was a positive trend between the recipient to transmitter α4β7 high CD8+ T cells binding ratio and days post infection (ρ = 0.60, p = 0.10) (Figure 6B).

We further compared recipient versus transmitter envelope virus replication in RA exposed flow cytometry confirmed CD4+ T cells expressing high cell surface levels of the α4β7 receptor from 4 different blood donation volunteers. In 1 of the 4 blood donor’s cells, higher replication was observed among the transmitter as compared to recipient envelope viruses (p = 0.01). In aggregate, transmitter as compared to recipient viruses showed significantly greater replication in α4β7 high CD4+ T cells (median recipient to transmitter ratio 0.51, range 0.10 – 6.89, p = 0.01) (Figure 6C). There was, however, a negative correlation (ρ = -0.36, p = 0.34) between time post infection and recipient to transmitter replication ratio in α4β7 high CD4+ T cells, although it was not statistically significant (Figure 6D). The goodness of the fit was not significantly better assuming either a polynomial or exponential decay (data not shown).

## Discussion

The combined envelope analysis from couples examined in this study and a previous investigation confirm that there is a selection for envelopes with signature genotypes, such as smaller less charged envelopes that are more closely related to ancestral strains [12]. One possibility is that the infectious source, i.e. genital secretions, contains a limited number of HIV-1 variants that are enriched in the envelope genotypes commonly observed in newly infected subjects. Source fluid studies, however, have failed to confirm this hypothesis [60-70]. Excluding random chance and infectious source compartmentalization suggests that transmitted viruses possess unique envelope properties that provide an advantage during transmission. To assess this issue, we examined various envelope phenotypic properties. We showed that recipient in comparison to the transmitter envelopes demonstrated no significant difference in the ability to use lower levels of the CD4 or CCR5 receptor, and they also had similar fusion characteristics. Interestingly, our correlation with days post infection analysis showed that recipient viruses isolated close to transmission were more sensitive to CCR5 inhibition compared to those sampled further from the estimated acquisition time. This buttresses previous conclusions that viruses found early compared to those circulating during the chronic phase of infection require relatively higher CCR5 levels for infection [24,36,42-46]. Transmitter envelope viruses were better at replicating in CD4+ T cells and DC/LC – CD4+ T cell co-cultures compared to recipient envelope recombinants. Transmitter envelope viruses also demonstrated significantly greater replication and enhanced binding to CD4+ and CD8+ T cells expressing high levels of the gut homing integrin. Collectively, viruses found early after infection have unique envelope genotypic characteristics, and variants with these genotypes do not have enhanced replication in potential early target cells or dissemination from the initial site of invasion using the gut homing integrin.

The SIV – macaque animal model shows that exposure to high levels of infectious virus leads to multiple small foci of localized infections at the site of invasion [29]. Other highly susceptible cells, such as CD4+ T cells, are recruited to these infectious clusters promoting infection dissemination and systemic spread. Similar to the SIV – macaque model, HIV-1 infection may begin with one or a small number of infected cells, and spread from these foci to other susceptible targets may disseminate the infection. We examined infection in LCs because LCs project their dendrites over lumen exposed epithelia, and thus they are likely the first DC subset that encounters incoming HIV-1 [49-52]. Prior studies have documented that R5 but not X4 viruses can infect LCs [18,71-74]. This counter-selection against X4 viruses has not been observed in any other mucosal cells, such as CD4+ T cells or MDDCs. This strongly suggests that LCs may function as the gatekeeper determining which viruses establish a productive infection. In our studies, however, we found that transmitter as compared to recipient envelope viruses replicated more efficiently in skin derived LCs – heterologous CD4+ T cell co-cultures. It should be noted, however, that we obtained LCs from discarded breast skin. Newer studies suggest that mucosal LCs are phenotypically different from skin LCs. Murine models demonstrate that genital LCs have different ontogeny and receptor expression compared to skin derived LCs [75,76]. Human *ex vivo* vaginal tissue studies also show that lumen exposed genital LCs may not express langerin, which is a hallmark of skin derived LCs [77]. One study suggests that langerin traffics low levels of incoming HIV-1 away from a productive infection pathway towards degradation [78]. Thus, genital as compared to skin LCs may be inherently more susceptible to HIV-1. Furthermore, it has been suggested that genital LCs capture infectious virus and disseminate them to other susceptible target cells without being productively infected [77]. On the other hand, HIV-1 productively infects skin derived LCs, and infection can be blocked by specific receptor inhibitors [71,78-80]. In aggregate, skin derived LCs are not ideal surrogates for genital LCs. Infection studies have not been conducted with genital LCs because it has been difficult to isolate adequate numbers with sufficient purity. Future studies will need to examine if genital LCs dictate the observed genetic restriction during transmission.

Besides LCs, mucosal tissues also contain CD4+ T cells and other DC subsets, such as DC-SIGN + DCs. These cells, however, have limited direct access to the lumen within intact mucosa [49,50]. It is possible that LCs counter-select against X4 HIV-1, and the deeper lying cells preferentially select specific R5 variants from the diverse CCR5 using viruses present in the infectious source. We, however, found that transmitter as compared to recipient envelope viruses were better at replicating in CD4+ T cells and monocyte derived DC – T cell co-cultures, a surrogate for the DC-SIGN + DCs present in the mucosa. It has been demonstrated that DCs can capture virions and retain them in an infectious state for an extended period of time and then spread them to other permissive cells [80-85]. This trans infection pathway spreads HIV-1 more efficiently compared to cell-free virus infections. CD4+ T cells and DCs/LCs may still be some of the earliest cellular targets, but these cells likely do not dictate which variants circulating in the transmitting partner establishes a disseminated infection in the newly infected individual.

Disseminating from the initial infection focus could also influence which virus establishes a new infection in a naïve host. It has been speculated that attachment to the α4β7 integrin facilitates virus migration from mucosal sites to GALT, where high level replication occurs early after HIV-1 acquisition [31-33]. Indeed, some HIV-1 envelope surface subunits, gp120s, with transmission/early infection genotypes, such as shorter and less glycosylated variable loops, had higher binding to the α4β7 receptor compared to chronic phase gp120s [34,59]. We, however, found recipient as compared to transmitter envelope viruses demonstrated decreased attachment to CD8+ T cells and lower replication in CD4+ T cells expressing high levels of the α4β7 receptor, although this finding was not consistent among all the blood donor cells. This suggests that further studies on α4β7 utilization may be necessary to determine its exact role in transmission. In contrast to the previous study, we examined α4β7 interactions with envelope glycoproteins in the context of a virus particle and not with a gp120 envelope subunit [34]. Recent structural studies suggest that exposure of important envelope domains, such as the α4β7 binding site, potentially differs between a gp120 subunit compared to trimers on virus particles [86,87]. Although, our results suggest that enhanced α4β7 utilization may not provide a selective advantage during transmission, we did observe a non-significant negative correlation between replication in α4β7 high CD4+ T cells and days post-infection, suggesting that if this is a potential transmission phenotype it is lost relatively early after infection.

A recent study showed that full-length T/F as compared to chronic stage viruses have both enhanced cell free infectivity and MDDC usage potentially due to increased envelope expression [27]. In contrast to our investigation of subtype D HIV-1, they examined subtype B and C viruses. Because HIV-1 subtype D may have different phenotypic characteristics compared to the other clades, properties of the envelope variants found early after infection may be subtype dependent [88-91]. Most importantly, the T/F viruses were not compared to the variants circulating in the transmitting partner. Specific variants are acquired presumably because they possess a phenotypic property that confers fitness for transmission compared to the swarm circulating in the transmitting partner. This transmission phenotype may not necessarily distinguish all T/F viruses from unrelated chronic phase variants. Viruses present in a transmitter likely possess different phenotypic properties compared to those isolated from unrelated subjects sampled during the chronic phase of disease. Thus, comparing full-length T/F to unrelated chronic stage variants is not ideal. Future studies that use full-length molecular clones from recipient transmitter pairs may be better able to identify a virus property that confers an advantage during mucosal HIV-1 transmission.

Unlike prior investigations that compared full-length envelopes from recipient transmitter pairs or phylogenetically estimated T/F against unrelated chronic infection envelopes, we examined envelope properties in the context of a infectious clone as opposed to 293T derived single cycle virus pseudotypes [21-23,26]. Single cycle infections limit the ability to interrogate small replication differences in relatively impermissible cells, such as LCs/DCs. Furthermore, PBMC generated virus has different number of envelope spikes with distinct types of glycans compared to the virions produced from 293T transfections, which can influence receptor binding, neutralization and other properties [34,92,93]. Because glycan characteristics influence α4β7 binding and receptors present on LCs/DCs often interact with envelope carbohydrate moieties, PBMC generated viruses have more physiologically relevant phenotypes compared to the 293T transfection derived virions [34,94,95]. Although virus phenotypes can be altered among our PBMC passaged viruses, changes generally occur in long term cultures [96].

One of the primary limitations with our study is that we were not able to sample all the newly infected subjects at the earliest time after HIV-1 acquisition. Thus, we could not phylogenetically estimate the T/F sequence. Envelope gene modifications occur relatively early after HIV-1 acquisition [97,98]. These changes potentially affect envelope characteristics, and thus, viruses isolated early after acquisition may have different phenotypes compared to the transmitted strains. To partially address this concern, we examined correlations between the phenotype of interest and time post infection reasoning that a transient transmission property would demonstrate a significant negative correlation. Sensitivity to CD4 inhibitors and replication in α4β7 high CD4+ T cells showed a negative correlation although they were not statistically significant. Because we were not able to evaluate the T/F variant, we used bulk PCR to better recapitulate the properties of the virus swarm at the time of sampling. Although, bulk PCR has been associated with polymerase induced recombination changes, this strategy allowed us to compare the envelopes from the newly infected subjects to the diverse variants circulating in the chronically infected transmitter and not just a small number of chronic infection strains [9]. We reasoned that if a transmission phenotype changed dramatically within the first year after acquisition, recipient and transmitter swarms should have similar characteristics. In contrast, we found that viruses found in recently infected subjects compared to those present in the transmitting partner had significantly lower infectivity and decreased binding. If enhanced replication capacity in primary cells and/or increased binding to the α4β7 integrin is the property that allows for the selection observed during transmission, then viruses with these potential transmission phenotypes must be selected against early after acquisition and subsequently enriched during the chronic phase of disease. Examination of longitudinally sampled viruses may help differentiate among these possibilities.

## Conclusion

Counter-selection against X4 variants and preferential acquisition of R5 viruses with signature genotypes strongly suggests that the genetic restriction observed during mucosal HIV-1 acquisition is not a stochastic process. Mucosal LCs likely prevent CXCR4 using viruses from establishing a new infection. The biological mechanism, however, that favors specific R5 variants amongst the complex CCR5 using quasispecies circulating in the chronically infected transmitting partner still remains unclear. Our study of viruses incorporating recipient and transmitter envelopes suggests that replication capacity in potential early target cells and α4β7 integrin usage likely do not confer fitness for transmission. Comparing virus properties of full-length molecular clones from transmission pairs in the most relevant cells, such as mucosal LCs could shed valuable insights about the selective bottleneck during transmission. Vaccine and microbicide strategies that specifically target the virus characteristic that confers a fitness advantage during acquisition may be especially efficacious in preventing transmission.

## Methods

### Subjects

We examined newly infected monogamous subjects with their epidemiologically linked heterosexual partner from the RCCS in the Rakai district of southwestern Uganda [99,100]. In the RCCS, serum was collected approximately every 10 months for HIV-1 antibody testing, and newly seropositive subjects’ previously seronegative sample was tested for HIV-1 RNA with a pooled viral load assay as previously described [101,102]. In seropositive incident subjects, the HIV-1 acquisition date was estimated as the midpoint between the last seronegative visit and the first HIV-1 antibody positive collection day. In seronegative individuals with HIV-1 RNA positive samples, acquisition was estimated as 17 days prior to sampling. This study was approved by human subjects review boards at the Uganda Virus Research Institute, the AIDS Research Subcommittee of the Ugandan National Council for Science and Technology, Johns Hopkins University, Brigham and Women’s Hospital, and Boston University Medical Center. All subjects provided written informed consent.

### Envelope amplification and analysis

HIV-1 RNA was isolated from around 100 ul of the serum samples, and RT-PCR was used to amplify a library of full-length envelope genes using previously described primers and amplification conditions [9]. For each subject, a minimum of four independent PCRs were pooled to generate a library of envelope genes from each serum sample. Pooled envelope amplifications were inserted into linearized pCMV-NL4-3-PBS→LTRΔGp160 plasmid using yeast gap-repair homologous recombination as previously described [36,103]. All recombinant NL4-3 clones containing a subject’s envelope genes were pooled to generate plasmids containing a library of the subjects’ envelopes (pCMV-NL4-3-PBS→LTR + Envs). Eight to 12 individual full-length envelopes were isolated and sequenced from each subject’s clones. The NL4-3 recombinants contained chimeric *Vpu, Tat,* and *Rev* genes because the full-length envelope overlaps with these accessory virus proteins [104]. All unique sequences reported in this publication have been submitted to Genbank (KF985982 - KF986146). Transmission among individuals within a partnership was confirmed by the observed clustering in ML phylogenetic analysis. For each couple, ML phylogenies were generated using Paup with parameters from FindModel best fit evolutionary model as described previously [12]. The ML trees were used to estimate a MRCA and the distance from each sequence to the MRCA. Average of pairwise distances was used to estimate genetic diversity. Recipient and transmitter sequence divergence was estimated as the average distance from the recipient or transmitter estimated ancestor respectively as described previously [12]. Different envelope segments amino acid lengths and PNGS were analyzed as previously described [12].

### Replication competent recombinant viruses

Viruses were generated by co-transfecting 293T cells with equivalent quantities of CMV-NL4-3-LTR→Gag4 and the library of recombinant NL4-3 with subject’s envelopes (pCMV-NL4-3-PBS→LTR + Envs) as previously described [36]. Supernatants were collected 48 to 72 hours after transfection. Supernatants from 293T cells were passaged for a maximum of 7 days in activated PBMCs to generate higher titer virus stocks. The number of infectious particles was estimated on TZM-bl cells as previously described [25,105].

### Inhibitor sensitivity

TZM-bl, U87/CD4/CXCR4 and U87/CD4/CCR5 cells, T-20, Maraviroc, and CD4 B4 monoclonal antibody were obtained through Research and Reference Reagent Program, Division of AIDS, NIAID, NIH [41,106,107]. Infection of TZM-bl cells in the absence and presence of two-fold serial dilution of the inhibitor was used to estimate the 50% inhibitory concentration (IC_50_) as previously described [24]. All reported IC_50_s are mean estimates from a minimum of 3 independent assays. Coreceptor usage was determined by monitoring p24 production in U87/CD4/CXCR4 and U87/CD4/CCR5 cells infected with 500 IP of each virus supernatant.

### Primary cells and infections

Peripheral blood mononuclear cells were isolated from HIV-1 negative blood donation volunteer’s buffy coats using Ficoll Hypaque density centrifugation. Monocytes were isolated from PBMCs using the percoll gradient method [108]. Primary human immature DCs were derived from monocytes, as described previously [109]. Briefly, monocytes were cultured in RPMI/10% FBS containing recombinant human GM-CSF (0.5 μg/ml; Leukine, Berlex) and recombinant human IL-4, 100 U/ml (BD Biosciences) for 6 days. Mature DCs were obtained by culturing immature DCs at day six of culture for two additional days in the presence of 100 ng/ml of ultra-pure *E. coli* LPS (Sigma). Primary human CD4+ and CD8+ T cells were isolated from monocyte depleted PBMCs using antibody conjugated magnetic beads (Miltenyi Biotech) according to manufacturer’s instructions. CD4+ T cells were activated with 2% phytohaemagglutinin (PHA) and 20 ug/ml recombinant human IL-2 (r-IL-2) for 2 days. LCs were obtained using previously described methods [57]. Briefly, normal human skin from reduction mammoplasties was acquired as discarded surgical tissue. The epidermis was mechanically separated from adipose tissue, and overnight dispase incubation was used to remove the dermis. Further trypsin digestion was used to extract individual cells. Immature LCs were obtained from the trypsinized epidermal cells by fractionating through a discontinuous OptiPrep density gradient. Langerhans cells were further purified from the epidermal cells using a magnetic CD1a microbead kit (Miltenyi Biotech) [57,58].

Around 2×10^6^ CD4+ T cells were exposed to 1,000 infectious particles in the presence of 20 ug/ml diethylaminoethyl(DEAE)-Dextran. After two hours, cultures were washed a minimum of three times. Around 0.5 × 10^6^ immature or mature DCs were independently exposed to 1,000 infectious particles. After three hours, DC cultures were washed a minimum of three times to remove unbound virus. Virus exposed DC infections were cultured either with or without autologous activated CD4+ T cells. Around 1 × 10^4^ skin LCs and were exposed to 5,000 to 25,000 infectious virus. Cultures were washed after 72 hours to remove unbound virus, and then co-cultured with heterologous activated CD4+ T cells. Around 50% of the culture supernatant was removed every 3 to 4 days and replaced with fresh media. Culture supernatants were assessed for p24 antigen content using an in house assay as previously described [110]. Infectious virus concentration was also estimated by infecting 1 × 10^4^ TZM-bl cells with 8 serial two-fold dilutions of supernatant culture starting at 50 ul. All infections were done in triplicate in a 96 well format. Two days post-infection, TZM-bls were examined for beta-galactosidase production using Galacto-Light Plus System (Applied Biosystems). A linear interpolated curve of the relative light units (RLUs) versus supernatant dilution was used to estimate RLU/ul. The AUC was generated from the RLU/ul from various days post infection. Primary cell infections were repeated a minimum of 4 times with cells from 4 different buffy coats or discarded surgical tissue.

### Replication in CD4+ and binding to CD8+ T cells expressing high α4β7 integrin levels

Both CD8+ and CD4+ T cells were activated with PHA, r-IL-2, and RA for 6 days. Around 1 × 10^6^ CD8+ and CD4+ T cells were exposed to 1 × 10^5^ infectious virus for 1 hour at 4°C in binding buffer (10 mM HEPES, 150 mM NaCl (HBS Buffer) buffer with 100 μM CaCl2 and 1 mM MnCl2). Cells were washed a minimum of 3 times to remove unbound virus. RNA was isolated from the CD8+ T cells using the QIAAMP Viral RNA kit (QIAGEN). HIV-1 copies were quantified using quantitative RT-PCR using previously described methods [111,112]. The CD4+ T cells were incubated at 37°C 5% C0_2_, and the infectious virus concentration in the culture supernatants was measured after 3 days as detailed above.

### Statistical analysis

Summary characteristics of recipient virus envelopes were compared to the transmitter envelope variants using the Wilcoxon rank-sum test. Aggregate comparisons of the multiple recipient and transmitter envelopes among the different couples were done using the Wilcoxon rank-sum test stratified by pair. Recipient to transmitter ratios were compared to the expected value of 1 using the one-sample Wilcoxon signed-rank test. Recipient to transmitter ratio comparisons were stratified by different volunteers cells during the aggregate assessments. Correlations were assessed using the non-parametric Spearman rank correlation. All p-values were based on a two-sided test. All statistical analyses were done with either Intercooled Stata version 8.0 (Stata Corporation, College Station, TX) or SAS version 8.2 (SAS Institute, Cary NC).

## Competing interests

The authors declare that they have no competing interests.

## Authors' contributions

VPC and BE constructed the viruses, conducted the replication and binding experiments and contributed to manuscript development. NC and PHN isolated the envelopes and performed the sequence analysis. SS and SJL did the statistical analysis. SJR, OL and DS were clinical site investigators. RHG and TQ contributed to data interpretation and manuscript development. MS designed the study, oversaw clinical and laboratory aspects of the study, analyzed and interpreted the data, and contributed to the manuscript development. All authors read and approved the final manuscript.

## Supplementary Material

Additional file 1: Figure S1 Compares genetic diversity among virus stocks and bacterial clones. **Figure S2-S4.** Show replication characteristics in primary cells. **Figure S5-S6.** Show phenotypic characteristics of Langerhans cells and alpha4 beta7 expression in primary T cells. Click here for file
