# Supplementary material for: HIV-1 envelope replication and α4β7 utilization among newly infected subjects and their corresponding heterosexual partners
Source: Retrovirology. 2013 Dec 26;10:162. doi: 10.1186/1742-4690-10-162 (PMC3883469; doi:10.1186/1742-4690-10-162)
Supplement: Additional file 1: Figure S1 — Compares genetic diversity among virus stocks and bacterial clones. Figure S2-S4. Show replication characteristics in primary cells. Figure S5-S6. Show phenotypic characteristics of Langerhans cells and alpha4 beta7 expression in primary T cells. [file 1742-4690-10-162-S1.ppt]

## Slide 1
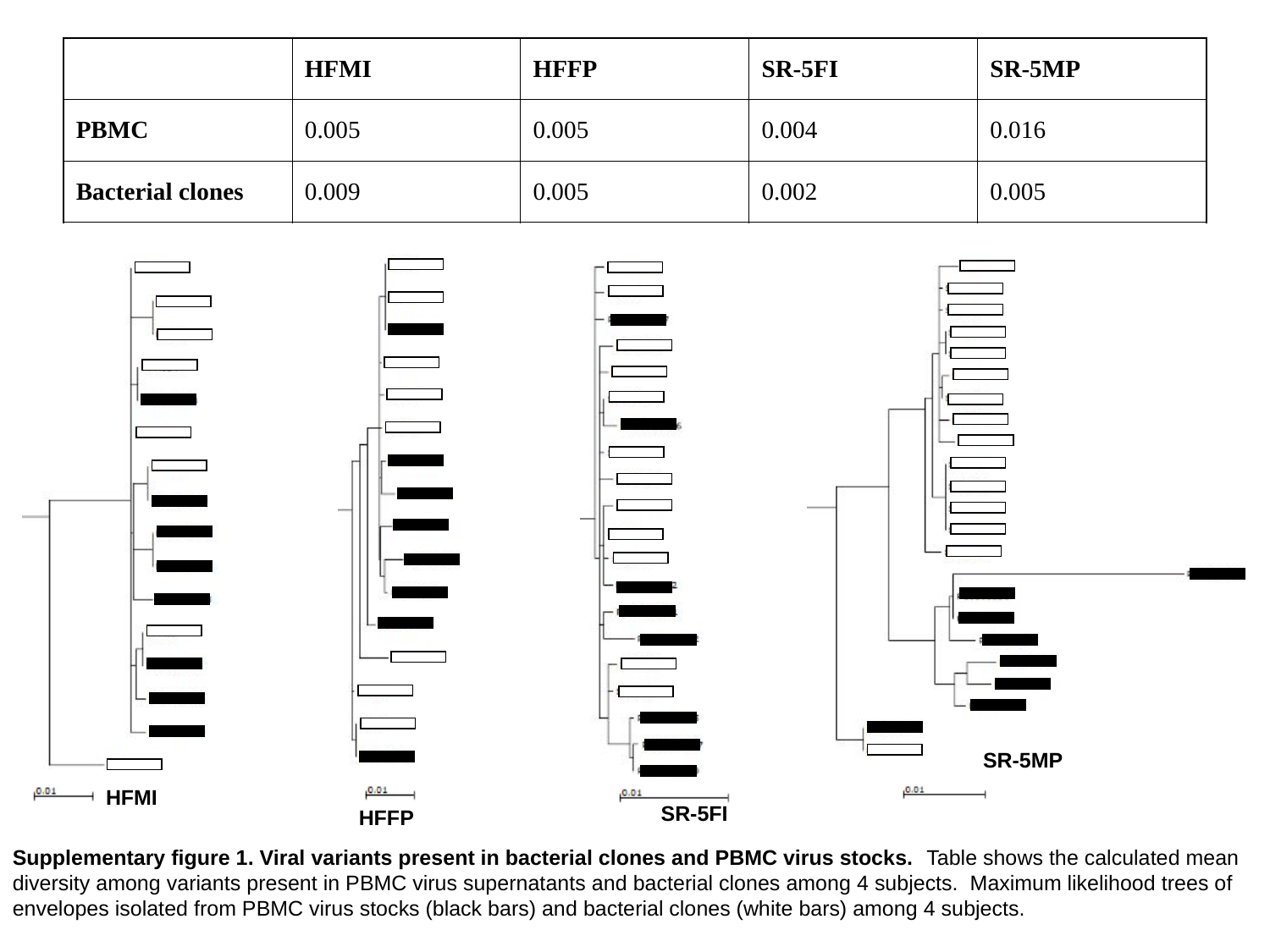

| | HFMI | HFFP | SR-5FI | SR-5MP |
| --- | --- | --- | --- | --- |
| PBMC | 0.005 | 0.005 | 0.004 | 0.016 |
| Bacterial clones | 0.009 | 0.005 | 0.002 | 0.005 |
SR-5MP
HFMI
SR-5FI
HFFP
Supplementary figure 1. Viral variants present in bacterial clones and PBMC virus stocks. Table shows the calculated mean diversity among variants present in PBMC virus supernatants and bacterial clones among 4 subjects. Maximum likelihood trees of envelopes isolated from PBMC virus stocks (black bars) and bacterial clones (white bars) among 4 subjects.

## Slide 2
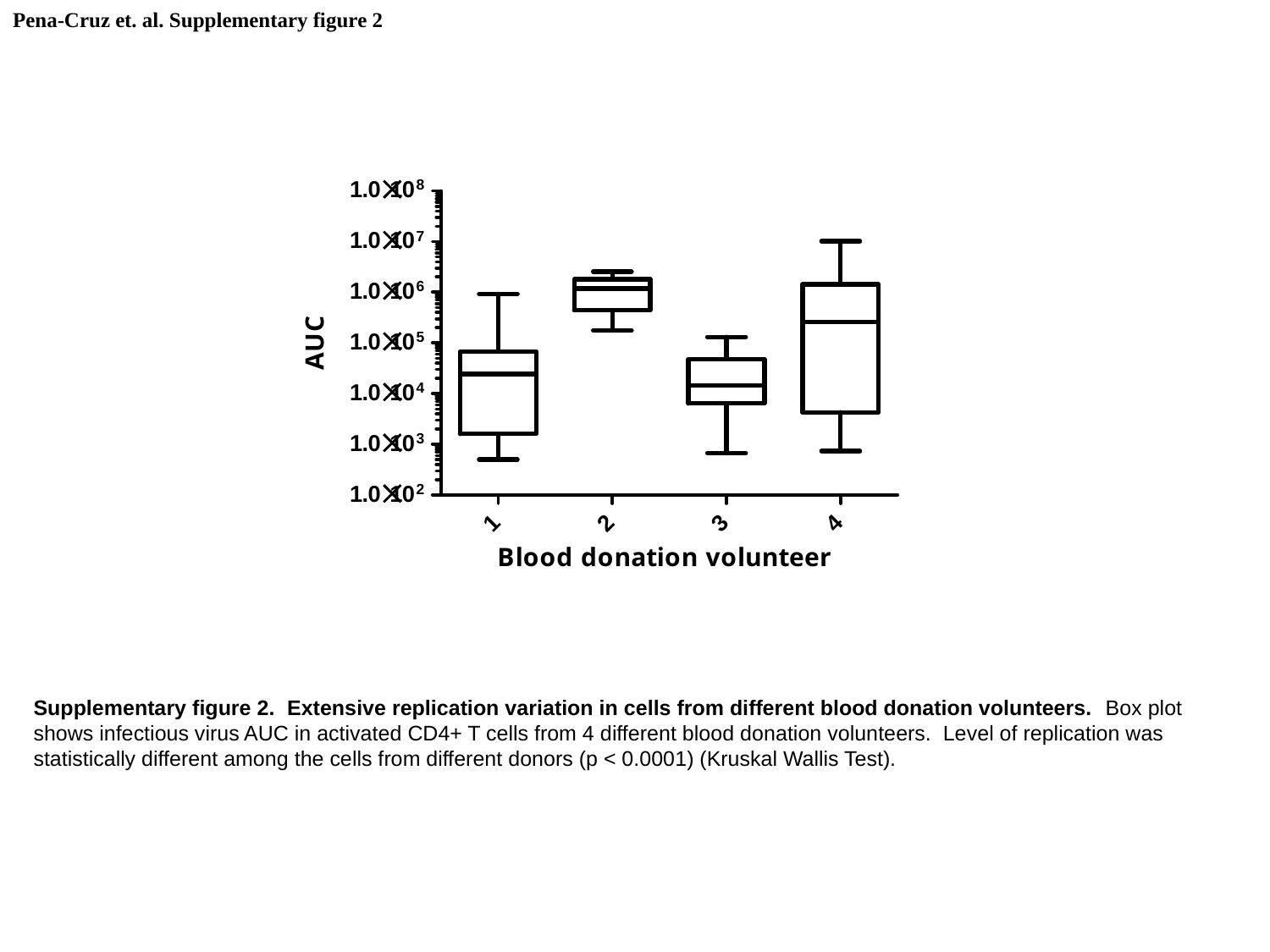

Pena-Cruz et. al. Supplementary figure 2
Supplementary figure 2. Extensive replication variation in cells from different blood donation volunteers. Box plot shows infectious virus AUC in activated CD4+ T cells from 4 different blood donation volunteers. Level of replication was statistically different among the cells from different donors (p < 0.0001) (Kruskal Wallis Test).

## Slide 3
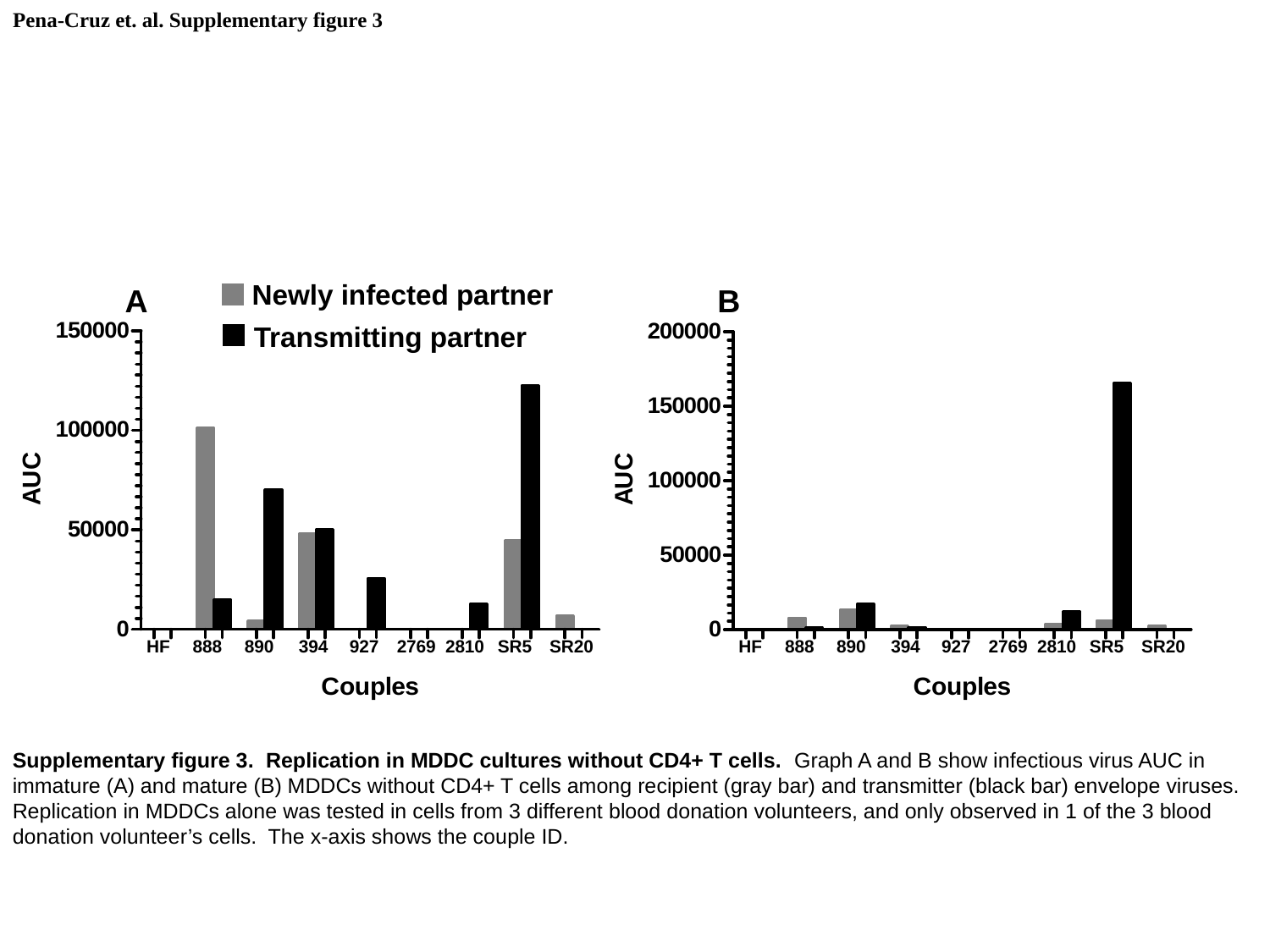

Pena-Cruz et. al. Supplementary figure 3
Newly infected partner
Transmitting partner
A
HF
888
890
394
927
2769
2810
SR5
SR20
B
HF
888
890
394
927
2769
2810
SR5
SR20
Supplementary figure 3. Replication in MDDC cultures without CD4+ T cells. Graph A and B show infectious virus AUC in immature (A) and mature (B) MDDCs without CD4+ T cells among recipient (gray bar) and transmitter (black bar) envelope viruses. Replication in MDDCs alone was tested in cells from 3 different blood donation volunteers, and only observed in 1 of the 3 blood donation volunteer’s cells. The x-axis shows the couple ID.

## Slide 4
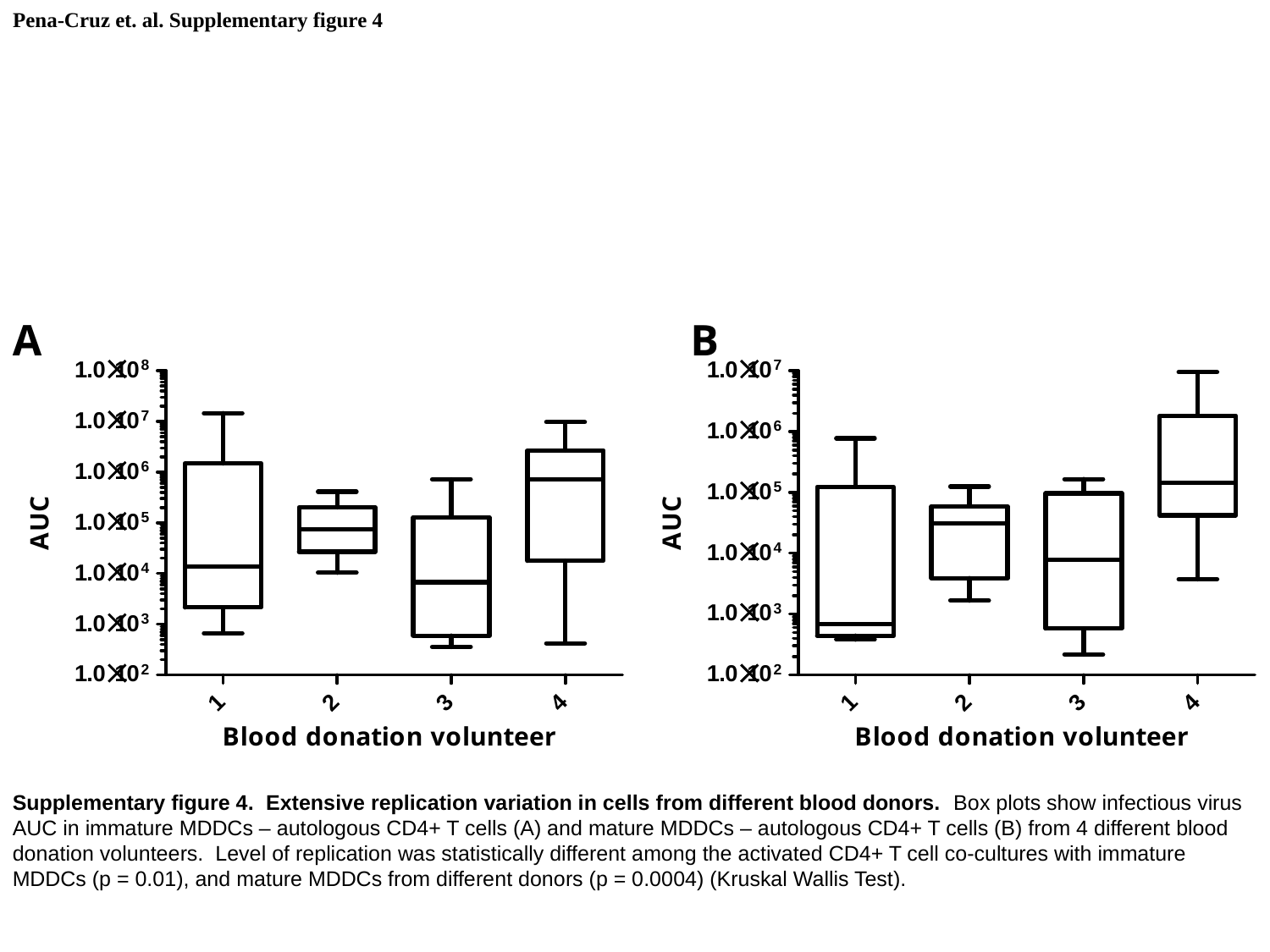

Pena-Cruz et. al. Supplementary figure 4
A
B
Supplementary figure 4. Extensive replication variation in cells from different blood donors. Box plots show infectious virus AUC in immature MDDCs – autologous CD4+ T cells (A) and mature MDDCs – autologous CD4+ T cells (B) from 4 different blood donation volunteers. Level of replication was statistically different among the activated CD4+ T cell co-cultures with immature MDDCs (p = 0.01), and mature MDDCs from different donors (p = 0.0004) (Kruskal Wallis Test).

## Slide 5
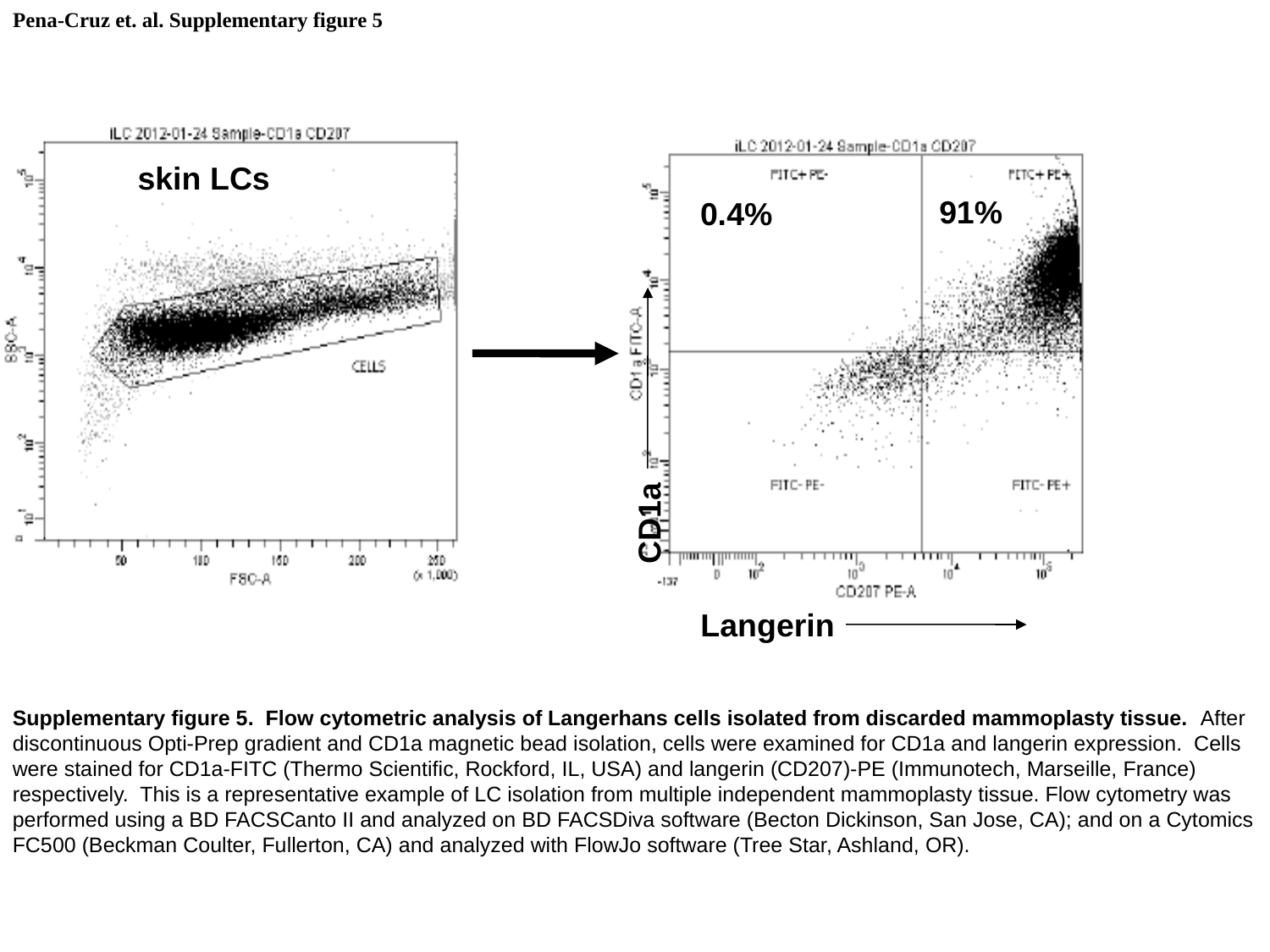

Pena-Cruz et. al. Supplementary figure 5
skin LCs
91%
0.4%
CD1a
Langerin
Supplementary figure 5. Flow cytometric analysis of Langerhans cells isolated from discarded mammoplasty tissue. After discontinuous Opti-Prep gradient and CD1a magnetic bead isolation, cells were examined for CD1a and langerin expression. Cells were stained for CD1a-FITC (Thermo Scientific, Rockford, IL, USA) and langerin (CD207)-PE (Immunotech, Marseille, France) respectively. This is a representative example of LC isolation from multiple independent mammoplasty tissue. Flow cytometry was performed using a BD FACSCanto II and analyzed on BD FACSDiva software (Becton Dickinson, San Jose, CA); and on a Cytomics FC500 (Beckman Coulter, Fullerton, CA) and analyzed with FlowJo software (Tree Star, Ashland, OR).

## Slide 6
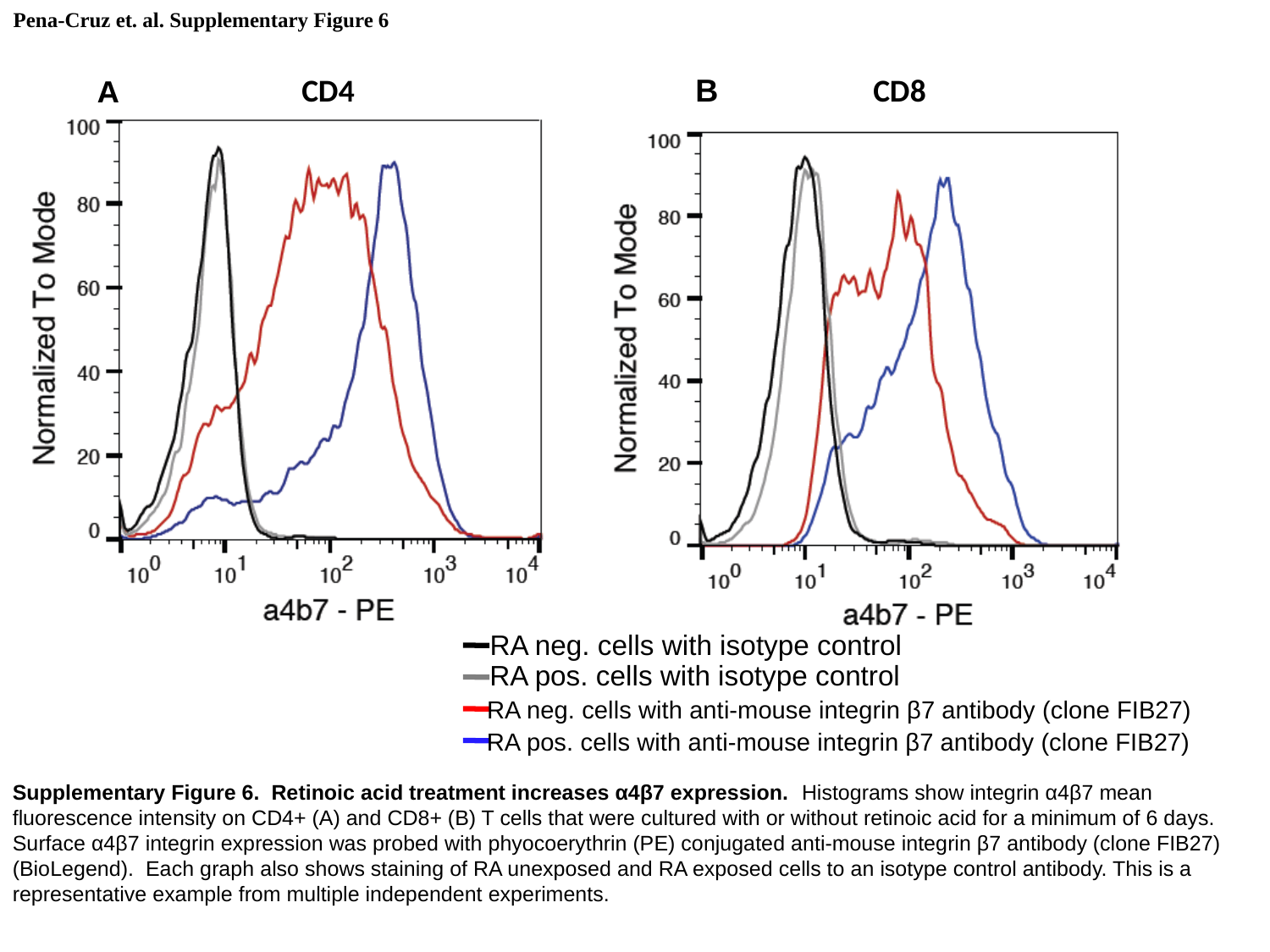

Pena-Cruz et. al. Supplementary Figure 6
CD4
CD8
B
A
B
RA neg. cells with isotype control
RA pos. cells with isotype control
RA neg. cells with anti-mouse integrin β7 antibody (clone FIB27)
RA pos. cells with anti-mouse integrin β7 antibody (clone FIB27)
Supplementary Figure 6. Retinoic acid treatment increases α4β7 expression. Histograms show integrin α4β7 mean fluorescence intensity on CD4+ (A) and CD8+ (B) T cells that were cultured with or without retinoic acid for a minimum of 6 days. Surface α4β7 integrin expression was probed with phyocoerythrin (PE) conjugated anti-mouse integrin β7 antibody (clone FIB27) (BioLegend). Each graph also shows staining of RA unexposed and RA exposed cells to an isotype control antibody. This is a representative example from multiple independent experiments.
